# CTC1‐STN1 coordinates G‐ and C‐strand synthesis to regulate telomere length

**DOI:** 10.1111/acel.12783

**Published:** 2018-05-17

**Authors:** Peili Gu, Shuting Jia, Taylor Takasugi, Eric Smith, Jayakrishnan Nandakumar, Eric Hendrickson, Sandy Chang

**Affiliations:** ^1^ Department of Laboratory Medicine Yale University School of Medicine New Haven Connecticut; ^2^ Lab of Molecular Genetics of Aging and Tumor Faculty of Medicine Kunming University of Science and Technology Kunming Yunnan Province China; ^3^ Department of Biochemistry, Molecular Biology and Biophysics University of Minnesota Medical School Minneapolis Minnesota; ^4^ Department of Molecular, Cellular, and Developmental Biology University of Michigan Ann Arbor Michigan; ^5^ Program in Chemical Biology University of Michigan Ann Arbor Michigan; ^6^ Department of Pathology Yale University School of Medicine New Haven Connecticut; ^7^ Molecular Biophysics and Biochemistry Yale University School of Medicine New Haven Connecticut

**Keywords:** DNA repair, stem cell aging, telomerase, telomere

## Abstract

Coats plus (CP) is a rare autosomal recessive disorder caused by mutations in CTC1, a component of the CST (CTC1, STN1, and TEN1) complex important for telomere length maintenance. The molecular basis of how CP mutations impact upon telomere length remains unclear. The CP CTC1^L1142H^ mutation has been previously shown to disrupt telomere maintenance. In this study, we used CRISPR/Cas9 to engineer this mutation into both alleles of HCT116 and RPE cells to demonstrate that CTC1:STN1 interaction is required to repress telomerase activity. CTC1^L1142H^ interacts poorly with STN1, leading to telomerase‐mediated telomere elongation. Impaired interaction between CTC1^L1142H^:STN1 and DNA Pol‐α results in increased telomerase recruitment to telomeres and further telomere elongation, revealing that C:S binding to DNA Pol‐α is required to fully repress telomerase activity. CP CTC1 mutants that fail to interact with DNA Pol‐α resulted in loss of C‐strand maintenance and catastrophic telomere shortening. Our findings place the CST complex as an important regulator of both G‐strand extensions by telomerase and C‐strand synthesis by DNA Pol‐α.

## 1 INTRODUCTION

The development of aging phenotypes is associated increased accumulation of damaged DNA in highly proliferative tissues, leading to compromised tissue homeostasis and frailty. Increasing evidence suggests that proper telomere function is critically important for the maintenance of a stable genome. Telomeres, protein:DNA complexes that cap the ends of chromosomes, play important roles in preventing the activation of DNA damage checkpoints that would otherwise induce cell cycle arrest and apoptosis. Mammalian telomeres consist of repeats of a TTAGGG lagging G‐strand and the complementary CCCTAA leading C‐strand sequences that end in a single‐stranded (ss) G‐rich overhang. Due to the inability of conventional DNA polymerases to copy the lagging‐strand of telomeric DNA, progressive telomere shortening occurs with each round of DNA replication in somatic cells. This “end replication problem” is solved by the enzyme telomerase, a specialized ribonucleoprotein complex that adds de novo telomere repeats to the 3′ G‐overhang. Telomerase is normally expressed only in human stem and progenitor cells. In somatic cells, the lack of telomerase expression results in progressive telomere shortening and reduced cellular lifespan. Consequently, overexpression of telomerase extends telomeres maintains genome stability and prevents the onset of replicative senescence.

Telomeres are bound by six telomere binding proteins, collectively termed Shelterin, which cap and protect telomeres (Palm & de Lange, 2008). The TTAGGG repeat binding factors, Telomere Recognition Factor 1 (TRF1) and Telomere Recognition Factor 2: Repressor/Activator binding Protein 1 (TRF2:RAP1), bind to the duplex portion of telomeric DNA. The Protection of Telomere 1 (POT1) protein interacts with the ss telomeric overhang and forms a heterodimer with TPP1 (a consensus name derived from the three competing acronyms TINT1, PTOP, and PIP1), while TRF1‐Interacting nuclear protein 2 (TIN2), the linchpin of this complex, bridges TPP1:POT1 with TRF1:TRF2:RAP1 (Hu et al., 2017). Shelterin components function to repress distinct DNA damage response and repair pathways at telomeres. For example, removal of TRF2 activates ATM to promote classical nonhomologous end joining (C‐NHEJ)‐mediated repair, while removal of TPP1:POT1 activates ATR and telomere repair via alternative (A)‐NHEJ‐mediated repair. Finally, RAP1 and TRF2 coordinate to repress the activation of homology‐directed DNA repair (Denchi & de Lange, 2007; Guo et al., 2007; Rai, Chen, Lei & Chang, 2016; Rai et al., 2017).

In addition to telomere end protection, Shelterin cooperates with multiple proteins to replicate telomeres. These proteins include the evolutionarily conserved CTC1: STN1: TEN1 (CST) complex. CTC1 and STN1 were originally discovered as proteins that stimulate DNA Pol‐α: primase activity, suggesting an essential role for CST in DNA replication (Casteel et al., 2009; Miyake et al., 2009; Surovtseva et al., 2009). Targeted deletion of CTC1 in the mouse, as well as depletion of individual CST components in human cells, all results in telomere replication defects and global telomere attrition, suggesting that the CST complex is required for multiple steps of telomere replication (Feng, Hsu, Kasbek, Chaiken & Price, 2017; Gu et al., 2012; Wu, Takai & de Lange, 2012). After DNA replication, leading‐strand telomeres are initially blunt‐ended, requiring nucleolytic processing of the leading‐strand termini to generate the 3′‐overhang needed for telomerase extension of the G‐strand. In contrast, the lagging‐strand telomeres already possess a 3′ G‐overhang amenable for telomerase extension. During S phase, TPP1 activates and stabilizes telomerase on the G‐overhang of both newly replicated leading‐ and lagging‐strand telomeres. However, telomere extension is restrained by the CST complex (Chen, Redon & Lingner, 2012). The recruitment of CST to telomeres, by POT1b in mouse cells and TPP1 in human cells, in turn promotes DNA Pol‐α to perform C‐strand fill‐in reactions (Wu et al., 2012). CST: DNA Pol‐α‐mediated C‐strand fill‐in is absolutely required for telomere length maintenance, as telomerase by itself is insufficient to generate the proper duplex telomere (Feng et al., 2017; Gu et al., 2012). Defects in telomere replication due to disruption of the CST complex lead to replication fork stalling, as telomeres can adopt secondary structures that are difficult to replicate (Gu et al., 2012; Stewart et al., 2012). Stalled replication forks and the failure of stalled fork restart at telomeres initiate aberrant homologous recombination events that in part account for the catastrophic loss of total telomeric DNA observed in mouse cells devoid of CTC1 (Gu et al., 2012), or the activation of a DDR in mammalian cells (Wang et al., 2012).

Missense mutations in the human CTC1 gene cause Coats plus (CP), a rare autosomal recessive disorder characterized by retinal telangiectasia, intracranial calcifications, osteopenia, and gastrointestinal bleeding (Anderson et al., 2012; Polvi et al., 2012; Walne et al., 2013). While some CP patients possess very short telomeres and have phenotypes resembling those patients with Dyskeratosis congenita (DC), suggesting that telomere maintenance is also functionally impaired in these patients, telomere lengths in other CP patients are not markedly reduced (Polvi et al., 2012). Biochemical characterizations revealed that most human CP mutations disrupted CST complex formation (Chen, Majerska & Lingner, 2013; Gu & Chang, 2013). One mutation, CTC1^L1142H^, is particularly interesting as it impacts on telomere length maintenance (Gu & Chang, 2013). CTC1^L1142^ is a highly conserved amino acid at the C‐terminus of the protein that plays a role in promoting STN1 interaction (Chen et al., 2013). As characterization of human disease mutations has often yielded valuable insights into basic biological functions, we investigated the impact of CTC1^L1142H^ on telomere metabolism. We compared the effect of this CTC1 mutation in two distinct cell types, the HCT116 colon cancer cell line and the telomerase‐immortalized retinal pigment epithelial (RPE) cells. We show that mutant CTC1^L1142H^ interacts poorly with STN1 and that the CTC1:STN1 subcomplex is sufficient to repress telomerase‐mediated telomere elongation. Expression of CP mutations that cannot interact with DNA Pol‐α show that CTC1:STN1 is also required to promote DNA Pol‐α‐mediated C‐strand maintenance. Our results reveal that the CST complex is required to coordinate both telomerase‐mediated G‐strand extension and DNA Pol‐α‐mediated C‐strand synthesis to maintain telomere length homeostasis.

## 2 RESULTS

### 2.1 Characterization of cells expressing the CTC1^L1142H^ mutation

**Figure 1:**
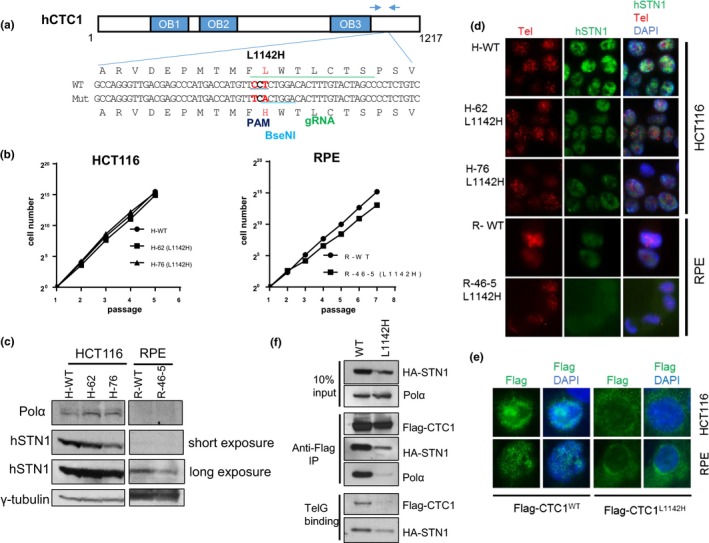
Generation of the CTC1 L1142H mutation in HCT116 and RPE cells using CRISPR/Cas9. (a) Schematic of the guide sgRNA utilized to mutate CTC1^L1142^ to CTC1^H1142^. Arrows indicate PCR primers used for genotyping. (b) NIH 3T3 assays were used to measure the proliferative capacities of the indicated cell lines. (c) Expression pattern of endogenous DNA Pol‐α and STN1 in the indicated cell lines detected by western analysis. γ‐tubulin was used as a loading control. (d) Immuno‐FISH analysis for endogenous STN1 (green) and telomeres (red) in WT or L1142H mutant HCT116 or RPE cell lines. STN1 was visualized using an anti‐STN1 antibody, telomeres visualized by hybridization with a 5′‐Tam‐OO‐(CCCTAA)_4_‐3′ PNA probe, and nuclei visualized by 4,6‐diamidino‐2‐phenylindole staining (DAPI; blue). (e) Immunostaining for WT Flag‐CTC1 or Flag‐CTC1^L1142H^ (green) expressed in HCT116 or RPE cells. Nuclei were stained with DAPI (in blue). (f) Co‐IP was used to determine the ability of WT Flag‐CTC1 and Flag‐CTC1^L1142H^ mutant proteins to interact with HA‐STN1, endogenous DNA pol‐α, and ss telomeric DNA [Tel‐G oligo: (TTAGGG)_3_]

Coats plus patients are compound heterozygous for two different *CTC1* mutations, with one allele harboring a frameshift mutation and the other a missense variant (Anderson et al., 2012; Keller et al., 2012; Polvi et al., 2012; Walne et al., 2013). Previous analysis of the human CTC1^L1142H^ mutation relied on transient expression of the mutant in HT1080 cells bearing wild‐type (WT) CTC1 alleles, making it difficult to understand the in vivo effects of this mutation (Chen et al., 2013). To understand mechanistically how the CTC1^L1142H^ mutation impacted telomere metabolism in CP patients, we utilized clustered, regularly interspaced, short palindromic repeats (CRISPR)/CRISPR‐associated 9 (Cas9) to mutate CTC1 Leu 1142 to His 1142 on both alleles in the HCT116 cell line and telomerase‐immortalized RPE cells (Figure 1a). A BseN1 restriction enzyme site was engineered into the targeted alleles to facilitate screening for correctly targeted cells (Supplementary Figure S1a, b), and Sanger sequencing confirmed correct mutagenesis (Supplementary Figure S1c). While CRISPR/Cas9‐mediated mutagenesis was highly efficient in HCT116 cell lines and yielded several correctly targeted clones, it was very difficult to generate CTC1^L1142H^ RPE mutants. We succeed in obtaining only one correctly targeted RPE CTC1^L1142H^ mutant cell line (Figure 1b). Analysis of two independently derived HCT116 CTC1^L1142H^ clones revealed that both grew at similar rates as the WT control and expressed DNA Pol‐α at similar levels (Figure 1b, c). Compared to WT controls, the CTC1^L1142H^ RPE clone R‐46‐5 exhibited slower growth after the first seven passages in vitro (Figure 1b). This reduced growth rate was likely not due to the activation of a DNA damage response at telomeres in CTC1^L1142H^ mutants, as we did not observe a significantly increased localization of the DNA damage signaling proteins γ‐H2AX and 53BP1 to telomere ends over WT controls (Supplementary Figure S1d, e). Western analysis showed that compared to WT controls, reduced STN1 level was observed in both cell types bearing the CTC1^L1142H^ mutation (Figure 1c). For both cell types, we attempted to detect the endogenous CTC1^L1142H^ mutant protein by immunofluorescence (IF) microscopy. However, a reliable antibody against endogenous CTC1 is not commercially available, and we were unsuccessful in our multiple attempts to generate antibodies against both human and mouse CTC1 (data not shown). To circumvent this difficulty, we performed IF microscopy using an anti‐STN1 antibody to visualize endogenous STN1, which we have shown previously to be a reliable marker to detect the endogenous CST complex (Gu et al., 2012). We found that STN1 is present exclusively in the nuclei of WT HCT116 cells, but in HCT116 CTC1^L1142H^ mutants, nuclear levels of STN1 are reduced (Figure 1d). Western analysis revealed that endogenous STN1 is present at low levels and was barely detectable in the nuclei of the RPE CTC1^L1142H^ mutant (Figure 1d). Expression of Flag‐CTC1^L1142H^ revealed both nuclear and cytoplasmic localization in HCT116 and RPE cells, suggesting that STN1:CTC1^L1142H^ interaction is unable to completely retain CTC1^L1142H^ to the nucleus (Figure 1e). Biochemical analyses revealed that Flag‐CTC1^L1142H^ displayed reduced ability to interact with both HA‐STN1 and DNA Pol‐α (Figure 1f). A DNA binding assay revealed that in the presence of HA‐STN1, Flag‐CTC1^L1142H^ also bound poorly to single‐stranded telomeric DNA (Tel‐G: TTAGGG_4_) (Figure 1f). Taken together, these results suggest that the CTC1^L1142H^ mutation disrupted interaction with STN1 and that STN1:CTC1^L1142H^ subcomplex cannot interact robustly with DNA Pol‐α or ss telomeric DNA, likely contributing to its partial localization to the cytoplasm. In addition, endogenous DNA Pol‐α levels are significantly higher in HCT116 tumor cells than in immortalized RPE cells.

### 2.2 Increased telomere length in CTC1^L1142H^ cells

**Figure 2:**
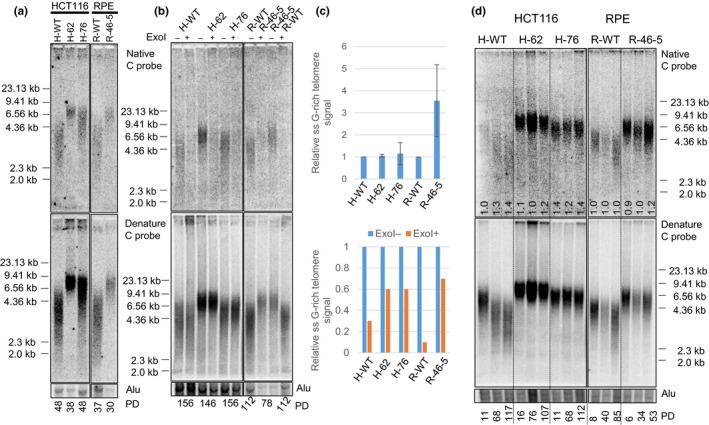
Increased telomere lengths in cells bearing the CTC1^L1142H^ mutation. (a) TRF Southern analysis of the lengths of single‐stranded (ss) (top panel) and total telomeric DNA (bottom panel) in cells of the indicated genotypes. Numbers at the bottom indicate the number of population doublings (PDs). (b) Telomere length analysis of single‐stranded (ss) (top panel) and total telomere length (bottom panel) in cells of the indicated genotypes either treated with (+) or without (−) ExoI. Alu was used as DNA loading control. (c) Quantification of the relative ss G‐rich telomere signal normalized to total telomeric signal in cells of the indicated genotypes, either untreated (top) or treated (bottom) with Exo I. Values represent the mean from three independent experiments and error bars represent standard error of the mean (SEM). (d) Telomere length analysis of single‐stranded (ss) (top panel) and total telomere length (bottom panel) of cells of the indicated genotypes subjected to long‐term serial passaging. PD: population doublings. Alu was used as DNA loading control. Numbers in native gel refer to ratio of overhang signal intensity to total telomere intensity

We next examined telomere length in WT and CTC1^L1142H^ mutant cells continuously passaged in culture, using telomere restriction fragment (TRF) Southern analysis and native in‐gel DNA hybridization with a CCCTAA (C‐probe) oligonucleotide complementary to TTAGGG to detect the 3′‐telomeric overhang repeats. Cells were harvested at the indicated population doublings (PDs) and genomic DNA prepared, digested with Hinf1/Rsa1 and resolved by gel electrophoresis. After signal capture, the gel was denatured and rehybridized with the C‐probe to determine the amount of total telomeric DNA present. An Alu probe was used as an internal loading control. Surprisingly, compared to WT controls, both HCT116 and RPE CTC1^L1142H^ mutant cell lines exhibited significant telomere length increases, from an average telomere length of ~3.5 to ~9.1 kb (Figure 2a). Interestingly, the heterogeneous telomere lengths normally observed in WT cells, ranging in size from ~2 to ~6 kb, became more restricted in mutant cells, spanning in most cases from ~6.3 to ~9.5 kb. To determine the status of the 3′‐overhang in these cells, we normalized the total telomeric signal with the 3′‐overhang signal. In addition, we also used Exo I digestion to distinguish single‐stranded (ss) telomeric G‐overhang from internal regions of ss telomeric DNA. While no appreciable increase in ss telomeric signal was detected in HCT116 CTC1^L1142H^ mutant cell lines, the RPE CTC1^L1142H^ mutant exhibited an ~3.5‐fold increase in ss telomeric DNA, largely stemming from a 7‐fold increase in Exo I‐resistant telomeric DNA (Figure 2b–d). An increased amount of Exo I‐resistant ss telomeric DNA was also observed in HCT116 CTC1^L1142H^ mutants over WT control (Figure 2b and c). This increased accumulation of internal stretches of ss telomeric DNA likely represented defects in lagging‐strand synthesis during DNA replication, as endogenous DNA Pol‐α is present at very low levels in CTC1^L1142H^ RPE cells and cannot be efficiently recruited by CTC1^L1142H^ to telomeres (Figure 1c, e).

We next examined whether the elongated telomere lengths in CTC1^L1142H^ mutants remained stable during continuous passaging. While telomere lengths decreased in WT HCT116 and RPE controls after continuous serial passages in vitro for over 4 months, suggesting that these cells possessed insufficient telomerase to maintain bulk telomere lengths, telomere lengths in both HCT116 CTC1^L1142H^ mutant cell lines remain stably elevated after continuously passaging for ~110 PD (Figure 2d). Interestingly, the RPE CTC1^L1142H^ mutant displaying slight telomere shortening after 34 PD, with increased heterogeneity observed in both the ss overhang and total telomere lengths by 53 PD (Figure 2d). These results suggest that CTC1, in complex with STN1, negatively regulates telomere length. While the CTC1^L1142H^ mutation led to an initial increase in telomere length in RPE cells, this increase in length cannot be stably maintained.

### 2.3 CTC1 leucine 1142 limits telomerase‐mediated telomere elongation

**Figure 3:**
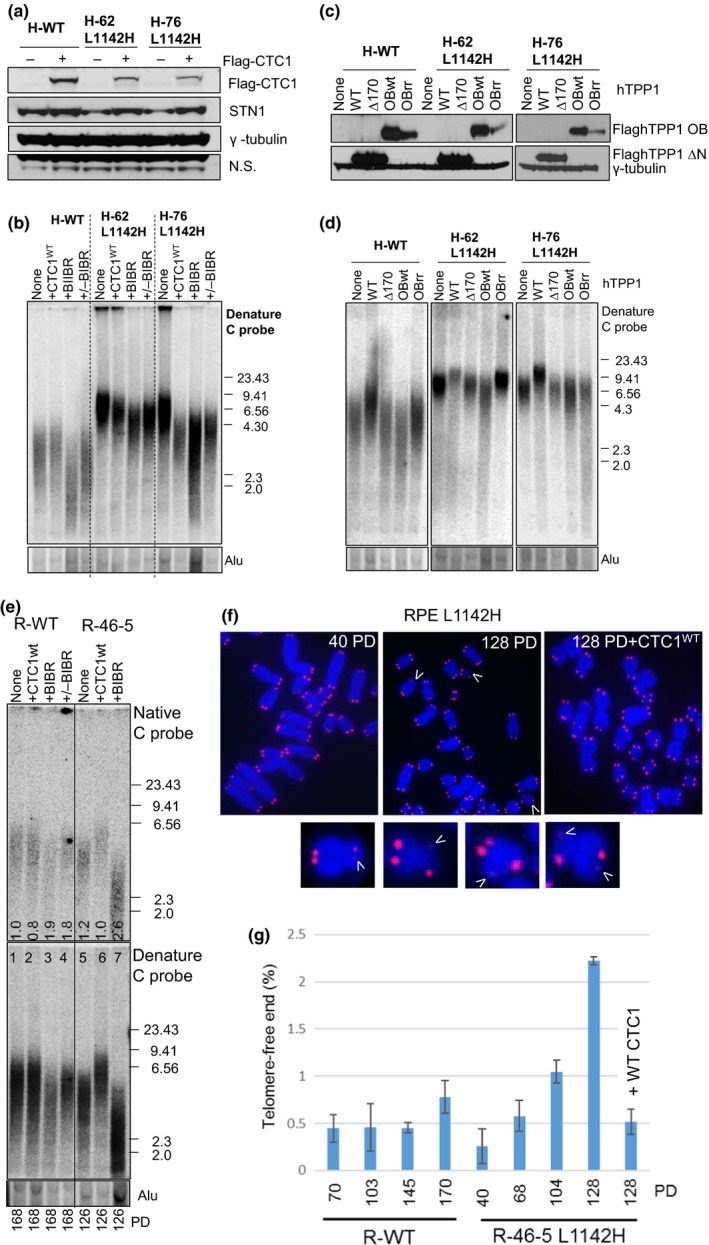
CTC1 L1142H promotes telomere elongation by telomerase. (a) Determination of the expression of WT Flag‐CTC1 and endogenous STN1 in the indicated cell lines by western analysis. (b) TRF Southern analysis of telomere length in WT or mutant HCT116 cells reconstituted with either WT CTC1 or cultured in the presence of 10 μM of the telomerase inhibitor BIBR. Cells were first passaged for 120 PD, reconstituted with WT CTC1 or treated with BIBR, and maintained for another 120 PD. +/−BIBR: cells were maintained in the presence of 10 μM BIBR for 60 PD, then maintained for another 60 PD after discontinuing BIBR treatment. (C) Expression levels of WT TPP1, TPP1∆170 or TPP1‐OB^WT^ or OB^RR^ (K166R; K167R mutations) in HCT116 cells by western analysis. (d) Analysis of total telomeric DNA in WT or mutant HCT116 cells expressing either WT TPP1, TPP1∆170 or TPP1‐OB^WT^ or OB^RR^. Cells expressed TPP1 constructs for 60 days before undergoing telomere length analysis by TRF Southern. Alu was used as a DNA loading control. (e) Telomere length analysis of single‐stranded (ss) (top panel) and total telomere length (bottom panel) in cells of the indicated genotypes. PD: population doublings. +CTC1: WT CTC1 was expressed for 120 days before the cells were harvested for telomere length analysis. +BIBR: cells were treated with 10 μM of the telomerase inhibitor BIBR. Numbers in native gel refer to ratio of overhang signal intensity to total telomere intensity. (f) Metaphase spreads revealing sister telomere loss in RPE^L^ ^1142H^ cells at the indicated PD. White arrowheads point to STLs. In one experiment, WT CTC1 was expressed in RPE^L^ ^1142H^ cells at PD 54 and then passaged for an additional 74 PD. (g) Quantification of the percentage of sister telomere loss in WT RPE or RPE^L^ ^1142H^ cells with the indicated PD when harvested

To understand mechanistically how telomere lengths increased in CTC1^L1142H^ mutants, we reconstituted WT Flag‐hCTC1 into WT and mutant HCT116 cell lines. Expression of WT Flag‐CTC1 increased endogenous STN1 levels in CTC1^L1142H^ cells, reinforcing the notion that endogenous STN1 is unstable in the presence of the CTC1^L1142H^ mutant (Figure 3a). Expression of WT Flag‐CTC1 decreased telomere length in HCT116 CTC1^L1142H^ mutant cell lines, in accord with previous observations that the CST complex normally represses telomere elongation (Figure 3b) (Chen et al., 2012). In contrast, WT Flag‐CTC1 had no impact on telomere length when expressed in WT HCT116 cells. We next tested whether telomerase is responsible for elongating telomeres in CTC1^L1142H^ mutants, using the telomerase inhibitor BIBR 1532. Treatment of both WT and HCT116 CTC1^L1142H^ cell lines with 10 μM BIBR 1532 resulted in rapid telomere shortening, while stopping BIBR treatment reversed this decline, resuming telomere elongation (Figure 3b). These results reinforce our observations that the CTC1^L1142H^ mutation is unable to restrain telomerase activity on the telomeric G‐strand, resulting in telomere elongation.

Telomerase recruitment to telomeres requires interaction with the oligosaccharide‐oligonucleotide binding (OB)‐fold domain of TPP1 (Nandakumar & Cech, 2012; Zhong et al., 2012). To examine whether expressing WT TPP1 in cells bearing the CTC1^L1142H^ mutation can lead to further extension of telomere length, we overexpressed WT TPP1, full length TPP1 bearing a single amino acid deletion in the acidic loop of the TEL patch (K170Δ) which abrogated its ability to interact with telomerase (Bisht, Smith, Tesmer & Nandakumar, 2016; Kocak et al., 2014; Nandakumar & Cech, 2012), WT TPP1‐OB‐fold domain, or TPP1‐OB‐fold domain containing two mutations that prevented association with telomerase (TPP1‐OB‐RR) (Zhong et al., 2012), in WT or CTC1^L1142H^ HCT116 cells (Figure 3c). After selection, cells were passaged for 60 days and telomere length was determined by TRF Southern. Expression of WT TPP1 resulted in telomere elongation in WT cells, from an average length of ~3.5 to ~4.5 kb. In CTC1^L1142H^ mutants, WT TPP further increased telomere length from an already long baseline level of ~6.5 to ~9.5 kb (Figure 3d). Telomere length did not increase further in both WT and CTC1^L1142H^ cells expressing TPP1‐Δ170, revealing that telomere length increase is due to TPP1‐mediated recruitment of telomerase. Compared to vector control, expression of WT TPP1‐OB, but not TPP1‐OB‐RR, led to rapid telomere shortening in both WT and CTC1^L1142H^ cell lines due to the competitive removal of telomerase from telomeres (Zhong et al., 2012). These results suggest that the CTC1^L1142H^ mutation is unable to repress telomerase recruitment by TPP1′s OB‐fold, resulting in further telomere elongation.

We next examined telomere lengths in telomerase‐immortalized RPE cells. TRF Southern reveal that R‐46‐5 mutant cells initially possessed long telomeres, but with increasing passages telomeres in this cell line shortened to the length of WT RPE cells (Figure 2d). Treatment of R‐46‐5 mutant cells with BIBR 1532 resulted in increased heterogeneity of the 3′ overhang and further shortening of both the overhang and total telomere length (Figure 3e). Telomere‐FISH revealed progressive increase in the percentage of sister chromatids with greatly reduced or missing telomere signals, a phenomenon termed sister telomere loss (STL), on metaphase spreads from late passage R‐46‐5 mutant cells, but not WT cells (Crabbe, Verdun, Haggblom & Karlseder, 2004) (Figure 3f, g). Fragile telomeres, prominent in CTC1 knockout mouse cells and suggestive of telomere replicative defects, were not significantly increased above background levels in CTC1^L1142H^ RPE mutants (data not shown) (Gu et al., 2012). While telomere length also shortened progressively in serially passaged WT RPE cells, sister telomere loss was infrequent and did not significantly increase with serial passages. Importantly, reconstitution of WT Flag‐hCTC1 into R‐46‐5 mutant cells prevented both progressive telomere shortening and sister telomere loss (Figure 3e–g), indicating that the CTC1^L1142H^ mutation directly contributed to the observed defects in telomere length maintenance.

### 2.4 Increased telomerase recruitment to telomeres in CTC1^L1142H^ mutants

**Figure 4:**
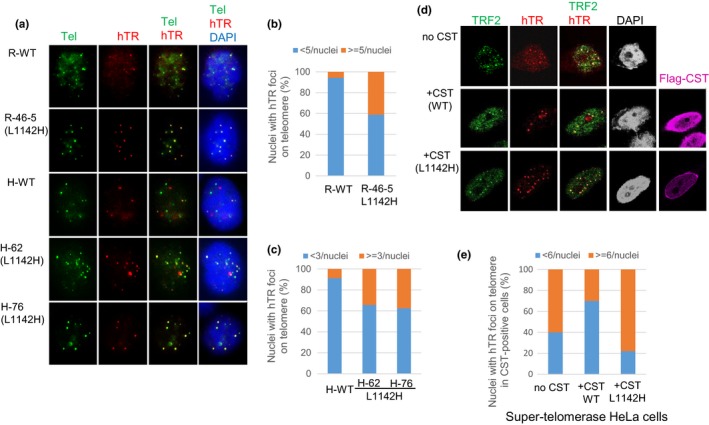
Increased telomerase recruitment to the telomeres of CTC1^L1142H^ mutant cells. (a) RPE or HCT116 cells were infected with retrovirus expressing hTERT and TPP1, then transiently transfected with hTR. Fluorescence in situ hybridization (FISH) was used to detect co‐localization of hTR (red) with telomeres (5′‐Tam‐OO‐(CCCTAA)_4_‐3′ PNA, green) in cells of the indicated genotypes. White arrows point to co‐localized hTR‐telomere signals in nuclei. (b, c) Quantification of the percentage of hTR‐positive foci on telomeres in RPE (b) or HCT116 (c) cells. At least 100 nuclei possessing co‐localized hTR signal on telomere were counted. (d) FISH was used to detect hTR foci (red), and immunofluorescence with anti‐Flag antibody was used to detect the Flag‐CST complex (purple) and a rabbit anti‐TRF2 antibody was used to detect endogenous TRF2 (green). Telomerase recruitment to telomeres is indicated in the merge panel by yellow spots. (Magnification: 100×). (e) Quantitation of the fraction of telomerase foci‐containing cells transfected with indicated CST constructs that contained ≤5 (blue) or ≥6 (orange) hTR foci per nucleus. Number of nuclei scored: WT CTC1: 55 nuclei, mutant CTC1: 67 nuclei, telomerase alone: 66 nuclei

To understand how the CTC1^L1142H^ mutation promotes telomere elongation, we performed telomerase FISH on WT and CTC1^L1142H^ mutant cell lines reconstituted with plasmids expressing hTERT (the catalytic component of telomerase), hTR (the RNA component of telomerase), and WT TPP1 (Kocak et al., 2014; Nandakumar & Cech, 2012). While only 5–10% of WT RPE and HCT116 cells displayed >3–5 hTR‐positive foci per nuclei, ~40% of CTC1^L1142H^ RPE cells displayed >5 hTR‐positive foci per nuclei. Similarly, ~40% of HCT116 CTC1^L1142H^ cells displayed >3 hTR‐positive foci per nuclei (Figures 4a–c, Supplementary Figure S2a, b). These results further support the notion that CTC1 represses telomerase localization to telomeres and that the CTC1^L1142H^ mutation alleviates this inhibition. We utilized a second approach to determine how the CTC1^L1142H^ mutation impacted telomerase recruitment to telomeres. HeLa cells transiently transfected with plasmids encoding hTERT, hTR, CTC1 (either WT or CTC1^L1142H^), WT STN1, and WT TEN1 were examined for telomerase recruitment to telomeres. A FISH probe against hTR was used to label telomerase, and a 5′‐Tam‐OO‐(CCCTAA)_4_‐3′ PNA probe was used to label telomeres. Under such overexpression conditions, the total number of observed hTR foci in the nucleus is a good indicator of the amount of telomerase recruitment. This is because successful telomerase recruitment to telomeres results in telomerase foci at several telomeres while lack of recruitment causes telomerase to reside in 1‐2 Cajal bodies in the nucleus. Only 22% of cells expressing WT CTC1 showed >6 hTR foci in the nucleus (Figure 4d, e). In contrast, 80% of cells expressing the CTC1^L1142H^ mutant displayed >6 hTR‐positive foci in the nucleus, indicating increased telomerase localization to telomeres in the presence of the CTC1^L1142H^ mutation.

### 2.5 CTC1 interaction with both STN1 and DNA Polymerase‐α is needed to completely repress telomerase‐mediated telomere length elongation

**Figure 5:**
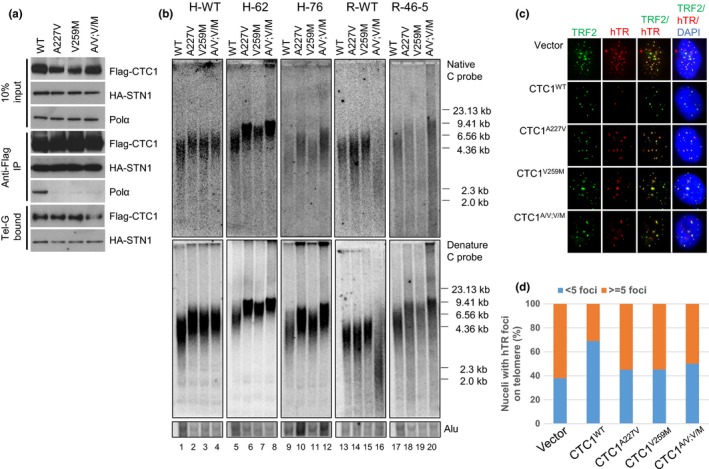
Disruption of CTC1:DNA Pol‐α interaction results in further telomere elongation in CTC1^L1142H^ mutant cells. (a) Biochemical characterization of Flag‐CTC1 WT and mutants unable to interact with endogenous DNA Pol‐α. Flag‐CTC1 was incubated with HA‐STN1 and interaction with endogenous DNA Pol‐α was determined by Co‐IP. (b) Analysis of the lengths of the 3′ overhang (top panel) and total telomere DNA (bottom panel) in HCT116 and RPE cells expressing WT CTC1, CTC1^A227V^, CTC1^V259M^, or CTC1^A227V, V259M^ mutants for 2 months by TRF Southern. Alu was used as a DNA loading control. (c) Expression of CTC1 mutants unable to interact with DNA Pol‐α increased telomerase recruitment to telomeres in CTC1^L1142H^ mutant RPE cells. Cells of the indicated genotypes were infected with retrovirus expressing hTERT and TPP1, then transiently transfected with a hTR cDNA. FISH was used to detect co‐localization of hTR (red) with telomeres (anti‐TRF2 antibody, green). (d) Quantification of (c). For each cell type, a minimum of 100 nuclei with signal were scored for the number of co‐localized foci

The observation that telomerase‐immortalized CTC1^L1142H^ RPE cells cannot maintain elongated telomere lengths over long passages and that expression of WT CTC1 prevented further telomere loss (Figure 3), suggest that telomerase‐mediated elongation of the G‐strand cannot fully maintain telomere lengths. We therefore examined how the C‐strand is maintained in CTC1^L1142H^ mutant cells. Previous research revealed that the CP mutations CTC1^A227V^ and CTC1^V259M^ abolished CTC1 interaction with DNA Pol‐α that also resulted in a paradoxical extension of telomere length (Chen et al., 2013). To understand how these CTC1 DNA Pol‐α interaction domain mutations impacted upon telomere length regulation, we generated CTC1^A227V^, CTC1^V259M^ and the CTC1^A227V; V259M^ double mutant and performed co‐immunoprecipitation (Co‐IP) experiments with DNA pol‐α (Figure 5a). While all three CTC1 mutants failed to interact with DNA pol‐α, they were still able to robustly bind to single‐stranded Tel‐G DNA in the presence of STN1. We next expressed WT CTC1, CTC1^A227V^, CTC1^V259M^, or CTC1^A227V; V259M^ in WT or CTC1^L1142H^ mutant cell lines (Supplementary Figure S3a). Compared to WT CTC1, expression of all three CTC1 DNA pol‐α mutants lead to an increase in both telomere length and G‐overhang in both WT and CTC1^L1142H^ HCT116 cells (Figure 5b). Expression of CTC1 DNA Pol‐α mutants, but not WT CTC1, also resulted in their enhanced localization to the nuclear periphery (Supplementary Figure S3b) and increased localization of telomerase to telomeres (Figure 5c and d). These results suggest that interaction of CTC1 with STN1 and DNA Pol‐α is required to fully repress telomerase activity.

As DNA Pol‐α is required for telomeric C‐strand fill‐in, which is a prerequisite for telomere length elongation, it is puzzling why expressing CTC1 DNA Pol‐α interaction domain mutants did not induce telomere shortening. We surmised that this was due to the presence of high levels of endogenous DNA Pol‐α in HCT116 cells (Figure 1c). We tested this hypothesis by expressing CTC1 mutants unable to interact with DNA Pol‐α in RPE cells possessing low levels of endogenous DNA Pol‐α. While a slight increase in telomere length was observed when these mutants were expressed in CTC1^L1142H^ RPE cells, both the lengths of total telomeres and the 3′‐G‐overhangs were extremely heterogeneous, suggesting a defect in DNA Pol‐α‐mediated C‐strand fill‐in reaction (Figure 5b). It is important that, expression of the CTC1^A227V; V259M^ double mutant in WT RPE cells led to dramatic telomere loss and the disappearance of the 3′ overhang. In addition, a 2.5‐fold increase in the number of STLs and a 6‐fold increase in the number of fragile telomeres, indicative of telomere replication defects, were observed (Supplementary Figure S4a–c). We surmised that expression of the CTC1^A227V; V259M^ mutant severely disrupted the localization of endogenous DNA Pol‐α to the C‐strand of telomeres. Coupled with the inability of telomerase to elongate telomeres due to the presence of endogenous WT CTC1, catastrophic telomere shortening generated a phenotype reminiscent to what was observed in CTC1 null mouse cells (Gu et al., 2012).

### 2.6 The CTC1‐STN1‐DNA Pol‐α complex inhibits telomerase recruitment to telomeres

**Figure 6:**
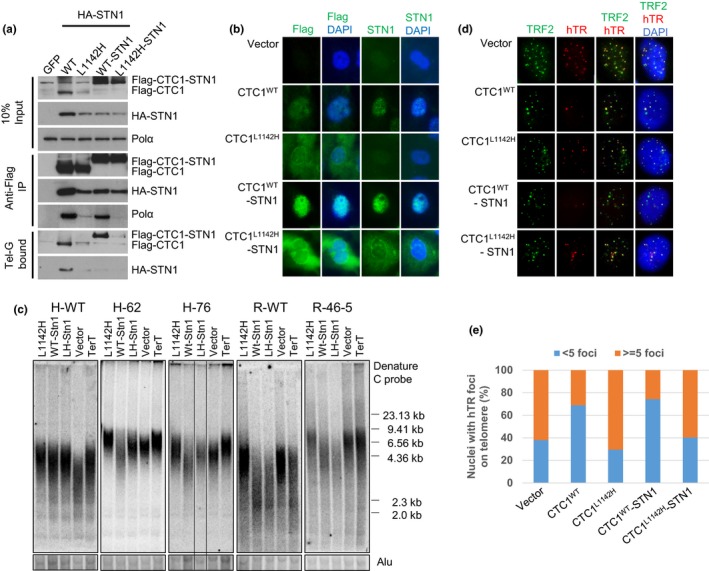
The CTC1:STN1 complex inhibits telomerase recruitment to telomeres. (a) WT Flag‐CTC1, Flag‐CTC1^L1142H^, WT Flag‐CTC1 tethered to STN1 or Flag‐CTC1^L1142H^ tethered to STN1 were examined for their ability to interact with HA‐STN1, endogenous DNA Pol‐α and ss Tel‐G oligo. (b) IF examination of the cellular distribution of WT Flag‐CTC1, Flag‐CTC1^L1142H^, WT Flag‐CTC1‐STN1, or Flag‐CTC1^L1142H^‐STN1 in CTC1^L1142H^ mutant RPE cells using anti‐Flag antibody (green). Blue: DAPI staining to detect nuclei. (c) TRF Southern analysis of telomere lengths in WT or CTC1^L1142H^ mutant HCT116 or RPE cells expressing the indicated DNA constructs for 2 months. Alu was used as DNA loading control. (d) Tethering CTC1^L1142H^ to STN1 does not inhibit telomerase recruitment to telomeres in CTC1^L1142H^ mutant RPE cells. Cells of the indicated genotypes were infected with a retrovirus expressing hTERT and TPP1, then transiently transfected with a hTR cDNA. FISH was used to detect co‐localization of hTERC (red) with telomeres (anti‐TRF2 antibody, green). (e) Quantification of (d). A minimum of 100 nuclei for each cell type bearing hTR signals were scored for co‐localization of telomerase with telomeres

The CTC1^L1142H^ mutant interacts poorly with both STN1 and DNA Pol‐α and fails to bind ss telomeric DNA, suggesting that physical interactions between CTC1, STN1, and DNA Pol‐α are all required to bind to ss telomeric DNA. To test this hypothesis, we examined whether artificially tethering mutant Flag‐CTC1^L1142H^ to STN1 via a flexible 10‐amino acid linker could rescue CTC1^L1142H^'s interaction with DNA Pol‐α and ss telomeric DNA. The Flag‐CTC1^WT^‐linker‐STN1 protein interacted robustly with both DNA Pol‐α and ss telomeric DNA (Figure 6a), completely localized to the nucleus (Figure 6b) and functionally reduced telomere lengths in both WT and CTC1^L1142H^ HCT116 and RPE cells (Figure 6c). In addition, expression of the Flag‐CTC1^WT^‐linker‐STN1 constructs reduced telomerase accumulation on telomeres, as revealed by telomerase FISH (Figure 6d and e). In contrast, while tethering STN1 to Flag‐CTC1^L1142H^ increased the expression levels of Flag‐CTC1^L1142H^, the Flag‐CTC1^L1142H^‐linker‐STN1 construct was unable to interact with either DNA Pol‐α or ss telomeric DNA, and it still localized partially to the cytoplasm (Figure 6a, b). Flag‐CTC1^L1142H^‐linker‐STN1 also dramatically reduced telomere lengths in both WT and CTC1^L1142H^ RPE cells, likely by functioning as a dominant negative to reduce endogenous DNA Pol‐α accumulation at telomeres (Figure 6c). These results suggest that CTC1 is required to directly interact with STN1 to form a CTC1:STN1 (C:S) complex. C:S then interacts with DNA Pol‐α to enable stable binding to ss telomeric DNA, and this C:S:DNA Pol‐α complex is inhibitory to telomerase‐mediated G‐strand extension.

### 2.7 TEN1 promotes CTC1:STN1:DNA Pol‐α complex formation

**Figure 7:**
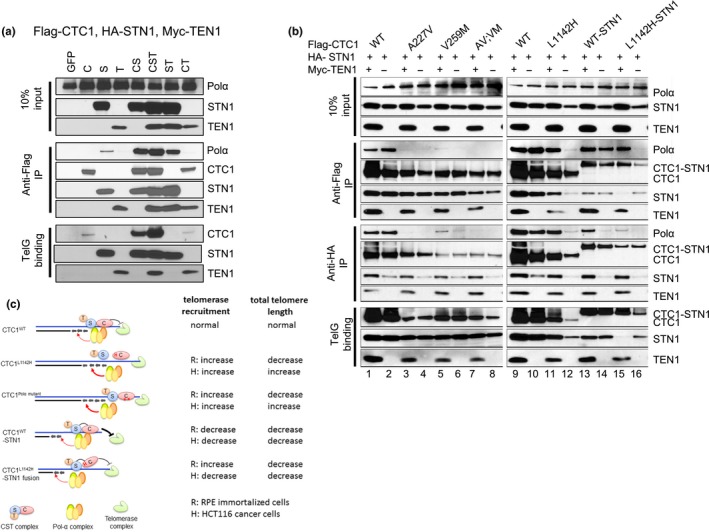
TEN1 enhances CTC1:STN1 interaction. (a) Biochemical characterization of Flag‐CTC1, HA‐STN1, and Myc‐TEN1 interaction with endogenous DNA Pol‐α and ss Tel‐G oligo. (b) Characterization of protein interactions between WT Flag‐CTC1, Flag‐CTC1 mutants, HA‐STN1, with (+) or without (−) Myc‐TEN1, with endogenous DNA Pol‐α and ss Tel‐G oligo. (c) Summary of how WT and CTC1 mutants interact with DNA Pol‐α to influence telomere binding and telomere length maintenance

To examine the contribution of TEN1 to CST complex formation with DNA Pol‐α and ss telomeric DNA, we first expressed Flag‐CTC1, HA‐STN1, and Myc‐TEN1 in HEK293T cells and examined their interactions by Co‐IP. By itself, HA‐STN1, but not Flag‐CTC1 or Myc‐TEN1, weakly interacted with endogenous DNA Pol‐α (Figure 7a). Co‐expressing all three C:S:T components together resulted in robust binding to DNA Pol‐α, although C:S and S:T also interacted well with DNA Pol‐α. The presence of Myc‐TEN1 enhanced the interaction between Flag‐CTC1^L1142H^ and HA‐STN1, as well as complex formation between Flag‐CTC1^L1142H^, HA‐STN1, and DNA Pol‐α (Figure 7b). TEN1 also promoted the interaction between Flag‐CTC1^L1142H^‐linker‐STN1 with endogenous DNA Pol‐α and ss DNA (compare Figures 6a to 7b). In addition, TEN1 also promoted the interaction between Flag‐CTC1^L1142H^, HA‐STN1, DNA Pol‐α, and ss telomeric DNA. These results suggest that the trimeric CST complex is required to efficiently interact with both DNA Pol‐α and ss telomeric DNA.

## 3 DISCUSSION

The CST complex has emerged as a negative regulator of telomerase‐mediated telomere elongation (Chen et al., 2012). Deletion of CTC1 results in extensive G‐overhang extension due to increased synthesis by telomerase. However, it is unclear how individual CST components function to regulate telomere length. As the CST complex also participates in a myriad of other biological activities at nontelomeric genomic sites, including the restart of stalled replication forks at GC rich loci (Chastain et al., 2016), we reasoned that deleting individual CST components will likely lead to confounding effects not associated with telomere length regulation. To circumvent this issue, we used CRISPR/Cas9 to engineer the CTC1^L1142H^ CP point mutation into both alleles of HCT116 and RPE cell lines. This mutation has been previously shown biochemically to reduce the interaction between CTC1 and STN1 and to promote telomere elongation (Chen et al., 2013; Gu & Chang, 2013). We provide functional evidence that CTC1:STN1 is required to repress telomerase activity in vivo. The CTC1^L1142H^ protein interacts poorly with STN1 and localizes partially to the cytoplasm, leading to telomerase‐mediated telomere elongation (Figure 7c). Biochemical characterization revealed that CTC1^L1142H^, STN1, TEN1 interact poorly with both DNA Pol‐α and telomeric DNA, suggesting that the CTC1^L1142H^:STN1:TEN1 complex cannot compete with telomerase for access to the 3′ G‐rich overhang. Impaired interaction between CTC1^L1142H^:STN1 and DNA Pol‐α results in increased telomerase recruitment to telomeres and further telomere elongation, revealing that C:S binding to DNA Pol‐α is required to fully repress telomerase activity. In addition, we also show that C:S regulates C‐strand fill‐in by DNA Pol‐α. Our findings place the CST complex as the major regulator of both G‐strand extension and C‐strand fill‐in reactions.

Deletion of CTC1 in both mouse and human cells results in extensive G‐overhang extension due to both increased G‐strand synthesis by telomerase and defects in C‐strand fill‐in synthesis by DNA Pol‐α, suggesting that the CST complex coordinates both of these processes (Feng et al., 2017; Gu et al., 2012; Kasbek, Wang & Price, 2013; Wan, Qin, Songyang & Liu, 2009). In vitro experiments suggest that CST limits telomerase access to G‐rich telomeric DNA (Chen et al., 2012). Analysis of CTC1^L1142H^ mutant cells revealed that they possess elongated telomeres due to increased recruitment of telomerase to telomeres. Artificially tethering CTC1^L1142H^ to STN1 was unable to prevent telomere elongation, indicating that direct CTC1:STN1 interaction is required to impart negative regulation to telomerase. Our data indicate that CTC1 binding to STN1 regulates telomerase access to the G‐rich ss overhang and that TEN1 is dispensable in this process.

Even with abundant telomerase, failure to maintain the C‐strand results in significant telomere shortening over time. Telomerase‐immortalized RPE cells bearing the CTC1^L1142H^ mutation initially exhibited a rapid increase in telomere length due to unrestrained telomerase activity. However, telomeres did not remain stably elongated, exhibiting progressive shortening after extensive passaging, accompanied by increased heterogeneity of the 3′ overhang. Accumulation of internal ss G‐rich telomeric DNA in CTC1^L1142H^ mutant cells suggests defective maintenance of the C‐strand, which is further exacerbated by the low level of endogenous DNA Pol‐α present in RPEs. Furthermore, our data suggest that C:S also regulates C‐strand maintenance by DNA Pol‐α (Figure 7c). Both CTC1 and STN1 have been shown to interact with DNA Pol‐α, with a recent report suggesting that STN1 stimulates the switch between RNA priming and DNA synthesis activities of DNA Pol‐α (Ganduri & Lue, 2017; Huang, Dai & Chai, 2012; Nakaoka, Nishiyama, Saito & Ishikawa, 2012). Telomere shortening was further exacerbated with the introduction of dominant negative CTC1 mutants incapable of interacting with DNA Pol‐α, leading to marked heterogeneity in the length of the G‐overhang and a smear of very long telomeres suggestive of telomere hyperextension (Figure 6b). Defective C‐strand synthesis resulted in telomere loss manifested as STL. When endogenous DNA Pol‐α's ability to interact with telomeres is further compromised by expressing the dominant negative CP CTC1^A227V; V259M^ mutant that cannot bind to DNA Pol‐α, total telomere loss and the complete disappearance of the 3′ G‐overhang were observed. In addition, elevated cytogenetic defects including STL and fragile telomeres suggestive of telomere replication failure were observed. This catastrophic telomere shortening phenotype is reminiscent of the dramatic loss of telomere sequences observed in CTC1 null mice (Gu et al., 2012). Our data support a recent model of telomere maintenance linking DNA replication to telomere length regulation, with the CST complex regulating both G‐strand extension by telomerase and C‐strand fill‐in by DNA pol‐α (Greider, 2016).

Progressive telomere shortening was not observed in CTC1^L1142H^ mutant HCT116 tumor cells, revealing that C‐strand fill‐in synthesis is not negatively impacted in this cancer cells due to elevated levels of DNA Pol‐α. Introduction of dominant negative CTC1^A227V; V259M^ mutant into the CTC1^L1142H^ mutant background resulted in further telomere elongation, suggesting that CTC1 (and the CST complex) cooperates with DNA Pol‐α to negatively regulate telomerase. These findings are reminiscent of observations revealing that disrupting the interactions between CDC13 and DNA Pol‐α in yeast and CTC1 and DNA Pol‐α in mouse cells both result in telomere elongation (Adams & Holm, 1996; Chen et al., 2013; Grossi, Puglisi, Dmitriev, Lopes & Shore, 2004; Qi & Zakian, 2000). Our data also highlight the significant differences in telomere length maintenance mechanisms between normal and cancer cells. While most investigations on telomere length maintenance mechanisms focus on the impact of telomerase, our findings suggest that the amount of endogenous DNA Pol‐α is an equally important consideration when evaluating telomere length maintenance mechanisms in normal and cancer cells, a finding likely to have important implications in aging research.

Because a subset of CP patients displays markedly shortened telomeres, this disease has been classified as a telomeropathy (Armanios & Blackburn, 2012). However, unlike the classical telomere shortening phenotype observed in DC patients, which is clearly due to defects in telomerase, not all CP patients display short telomeres (Polvi et al., 2012). Moreover, CP patients display clinical manifestations distinct from those observed in DC patients, suggesting that the underlying defects of these two diseases might be mechanistically distinct. Results gleaned from overexpression studies of human CP mutations in HCT116 cancer cells and expressing corresponding human CTC1 mutations into CTC1^−/−^ MEFs strongly suggest that CP is due to failure to properly maintain the telomeric C‐strand, leading to telomere replication defects (Chen et al., 2013; Gu & Chang, 2013; Gu et al., 2012). STL is a prominent feature in our late passage CTC1^L1142H^ mutant RPE cell lines, and this cytogenetic aberration has been previously found in cells lacking the RecQ helicase WRN, a protein necessary for the replication of G‐rich telomeric DNA (Crabbe et al., 2004). A recent report also suggests that C‐strand replication defects are associated with STLs (Takai et al., 2016). We speculate that in tissues bearing an elevated level of DNA Pol‐α, the elongated telomeres exhibited by the CTC1^L1142H^ mutation likely conferred an initial proliferative advantage. However, continuous cellular replication in tissues with limiting DNA Pol‐α levels results in C‐strand maintenance defects, manifested as stalled replication forks unable to bypass G‐rich secondary structures including G‐quadruplexes (G4), resulting in the formation of single‐stranded gaps that when degraded give rise to STL. Both POT1 and CST efficiently disrupt G‐quadruplex formation in vitro (Bhattacharjee, Wang, Diao & Price, 2017; Wang, Nora, Ghodke & Opresko, 2011), and our data suggest that introduction of WT CTC1 into CTC1^L1142H^ mutants completely suppressed STL formation (Figure 3g). We postulate that CST/POT1 plays an important role in preventing the formation of G4 on ss telomeric G‐rich DNA to maintain genome stability.

## 4 EXPERIMENTAL PROCEDURES

### 4.1 Plasmids and antibodies

CTC1 point mutations were generated by PCR. The fusion protein CTC1‐STN1 was linked by a 10 amino acids polyglycine spacer and a Flag‐tag was inserted at the N‐terminus of CTC1. The retrovirus vector pQXCIP (Clontech) was used for transient protein expression in 293T cells or stable expression in the HCT116 and RPE human cell lines. Antibodies that recognize phosphorylated γH2AX (Millipore #05‐636) and 53BP1 (Santa Cruz #sc‐22760) were used for the DNA damage assays. Mouse monoclonal anti‐TRF2 (Millipore #05‐521) or rabbit polyclonal anti‐TRF2 antibody (Novus NB110‐5713) was used to visualize telomeres for RNA‐FISH. The anti‐DNA Pol α antibody was purchased from Santa Cruz (#sc‐5921) and the anti‐STN1 antibody from Sigma (#WH0079991M1). Anti‐epitope tag antibodies were purchased from Sigma (anti‐Flag #F3165 and anti‐HA #A300‐305A) or Millipore (anti‐Myc #05‐724). The telomerase inhibitor BIBR1532 was purchased from Sigma.

### 4.2 Cell culture and the generation of CTC1‐L1142H mutant cell lines by CRISPR/Cas9

Human cancer HCT116 cells were maintained in McCoy's 5A media supplemented with 10% FBS. Human hTERT‐immortalized RPE cells were cultured in DMEM/F12 (1:1) media with 10% FBS. The CTC1‐L1142H point mutation targeting vector was constructed into rAAV‐vector GG‐MCS‐SEPT‐N2 (Kan, Batada & Hendrickson, 2017) and a new restriction enzyme site for BseNI was generated in the mutated site. The sgRNA was inserted into lentivirus plasmid px458 (containing Cas9:GFP) and was designed so that it would likely destroy the Hpy188III restriction enzyme recognition site around the mutation site. The targeting vector and sgRNA plasmids were co‐infected into HCT116 or RPE cells and targeted cell lines were screened by BseNI restriction enzyme digestion and further confirmed by DNA sequencing. To over‐express CTC1 wild‐type or mutant protein in HCT116 or RPE cells, the cells were infected by the relevant retrovirus and then selected for puromycin resistance for at least one week.

### 4.3 DNA binding and Co‐IP assays

Streptavidin‐sepharose beads (Invitrogen) coated with Biotin‐Tel‐G (TTAGGG)_6_ were used for the ss DNA binding assays. Antibody cross‐linked‐sepharose beads (Sigma) were used for Co‐IP. Both beads were incubated with crude cell lysates in TEB_150_ buffer (50 mM Hepes, pH 7.3, 150 mM NaCl, 2 mM MgCl_2_, 5 mM EGTA, 0.5% Triton X‐100, 10% glycerol and proteinase inhibitors) overnight at 4°C. After washing with same buffer, the beads were analyzed by immunoblot assay.

### 4.4 Immunofluorescence–fluorescence in situ hybridization

IF‐FISH experiments for telomerase recruitment in super‐telomerase HeLa cells were performed as previously reported (Bisht et al., 2016) with modifications to accommodate CST protein overexpression. Confluent six‐well plates containing HeLa cells were transfected with a 2:1:1 ratio of CTC1:STN1:TEN1and a 3:1 ratio of hTR:hTERT using lipofectamine 2000 (Life technologies) following manufacturer protocols. The total DNA transfected per well was held constant by complementation with empty vector if necessary and never exceeded 6 μg per well. Two days following transfection, cells were fixed with 4% formaldehyde in PBS for 10 min. Cells were washed with PBS and then permeabilized in a solution containing 0.5% Triton X‐100 and PBS. Following permeabilization, cells were blocked with PBS containing 1 mg/ml BSA, 3% goat serum, 0.1% Triton X‐100, 1 mM EDTA (pH 8.0) for 1 h. After washing with PBS, the cells were incubated with mouse monoclonal anti‐FLAG M2 (Sigma; F1804; 1:500) in combination with rabbit polyclonal anti‐TRF2 antibody (Novus NB110‐5713; 1:200) for 30 min. Alexa Fluor 488‐conjugated anti‐mouse IgG (Life Technologies) was used to detect FLAG‐tagged CST proteins by IF. Alexa Fluor 568‐conjugated anti‐rabbit IgG (Life Technologies) was used to detect endogenous TRF2.

RNA‐FISH assay in HCT116 or RPE cells was performed as follow. Cells were infected with hTPP1^∆N^, selected by puromycin, and transiently transfected with hTR:hTERT at a ratio of 3:1 for 2 days. Telomeres were visualized either by immunostaining with mouse monoclonal anti‐hTRF2 antibody and Alexa Fluor 488‐conjugated anti‐mouse IgG (Life Technologies) or by mixing 5′‐Fam‐OO‐(CCCTAA)_4_‐3′ probe with Cy5‐conjugated TR probe.

### 4.5 Metaphase PNA‐FISH and immunofluorescence (TIF) assays

To image metaphase spreads, cells were treated with 0.5 μg/ml of colcemid for 4 hr before harvest. Trypsinized cells were treated with 0.06 M KCl, fixed with methanol:acetic acid (3:1) and metaphases spread on glass slides. Metaphase spreads were hybridized with 5′‐Tam‐OO‐(CCCTAA)_4_‐3′ probe. For the TIF assay, cells were seeded in 8‐well chambers and immunostained with primary antibodies against γ‐H2AX or 53BP1, and then treated with FITC‐secondary antibodies before hybridization with the 5′‐Cy3‐OO‐(CCCTAA)_4_‐3′ probe to detect telomeres.

### 4.6 TRF Southern

Serial cultures were performed according to the 3T3 protocol as previously described (Blasco et al., 1997). To analyze telomere length, 20 μg of total genomic DNA was separated by 0.8% agarose gel electrophoresis. The gels were dried at 50°C and prehybridized at 55°C in Church mix (0.5 M NaH_2_PO_4_, pH 7.2, 7% SDS) and hybridized with γ‐^32^P‐(CCCTAA)_4_ oligonucleotide probes at 55°C overnight. The gels were washed with 4 × SSC, 0.1% SDS buffer at 55°C and exposed to phosphorimager screens. After in‐gel hybridization for the G‐overhang under native conditions, the gels were denatured with 0.5 N NaOH, 1.5 M NaCl solution and neutralized with 3 M NaCl, 0.5 M Tris‐HCl, pH 7.0, then re‐probed with γ‐^32^P‐(CCCTAA)_4_ oligonucleotide probes to detect total telomere DNA after denaturation. Finally, the gel was re‐denatured to remove all probe and rehybridized with a γ‐^32^P radiolabeled Alu probe (GTGATCCGCCCCGCCTCGGCCTCCCCAAAGTG) as an internal loading control. To determine the relative G‐overhang signals, the signal intensity for each lane was scanned with a Typhoon imager (GE) and quantified by ImageQuant (GE) before and after denaturation. The G‐overhang signal was normalized to the total telomeric DNA and compared between samples.

## CONFLICT OF INTEREST

The authors declare no competing financial interests.

## AUTHOR CONTRIBUTIONS

PG, EH, and SC conceived the project and designed the experiments; TT and EH generated and characterized mutant cell lines; PG and SJ performed telomerase FISH on CTC1 mutant cells and all the biochemistry and molecular biology experiments; and ES and JK performed telomerase FISH experiments on super‐telomerase HeLa cells expressing CTC1 mutants. PG, TT, SJ, ES, JK, EH, and SC analyzed and interpreted the data; PG and SC composed the figures and wrote the manuscript.
