# Supplementary figures and images for: CTC1‐STN1 coordinates G‐ and C‐strand synthesis to regulate telomere length
Source: Aging Cell. 2018 May 17;17(4):e12783. doi: 10.1111/acel.12783 (PMC6052479; doi:10.1111/acel.12783)

(a)

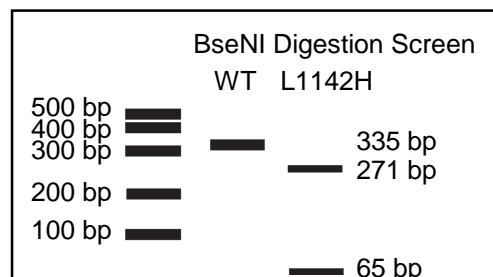

(b)

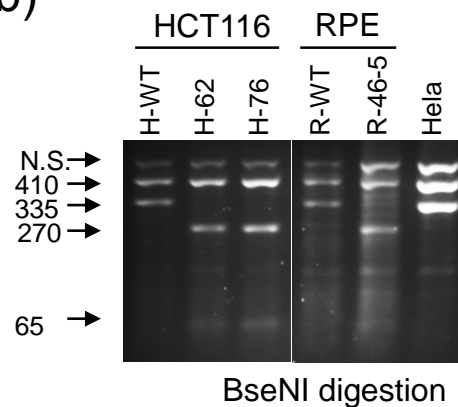

(c)

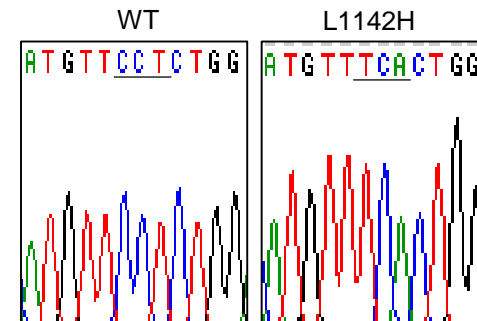

(d)

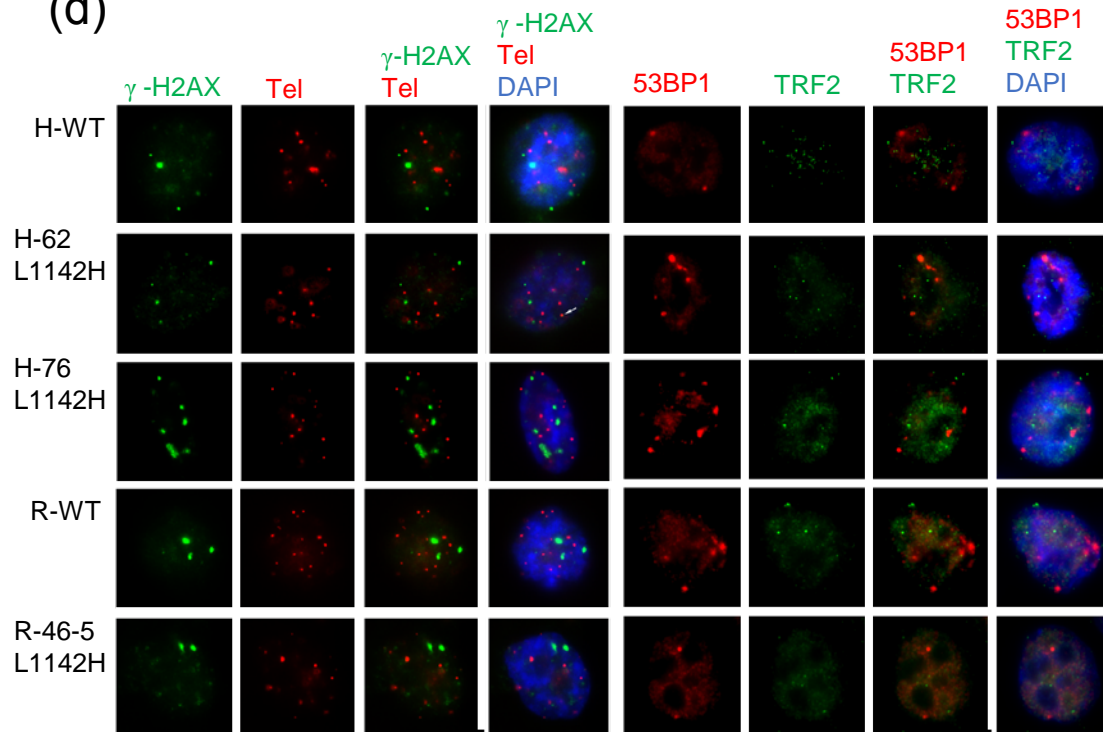

(e)

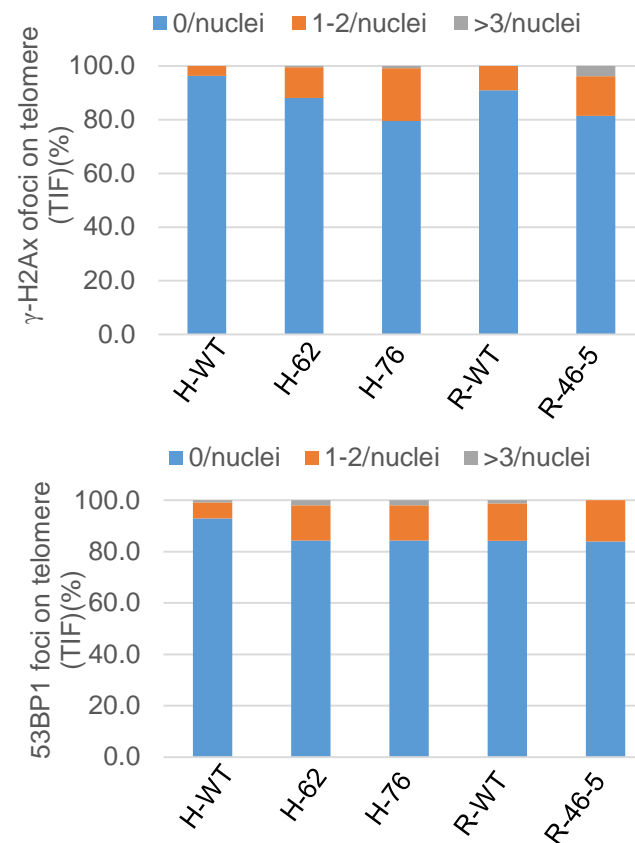

(a)

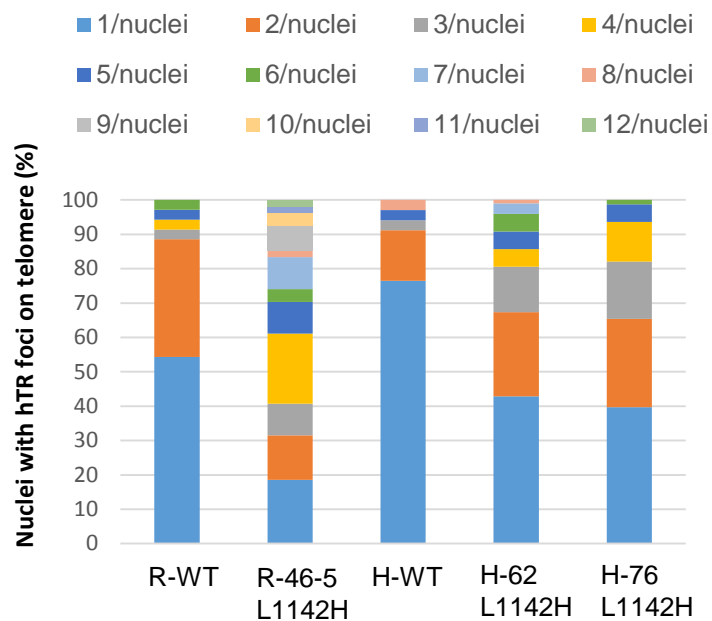

(b)

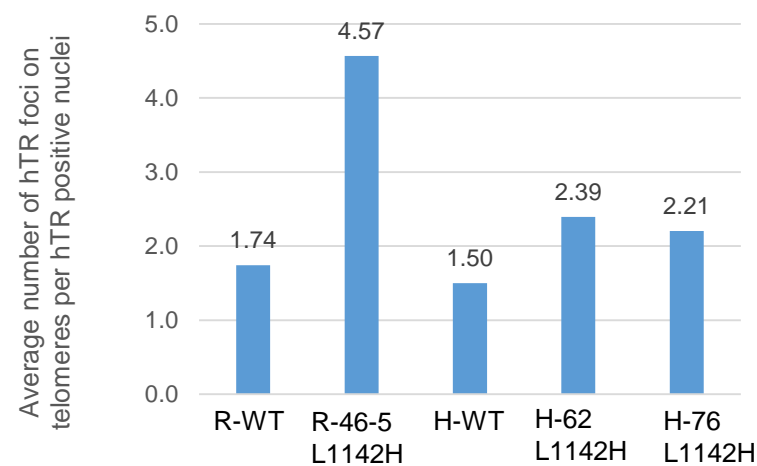

(a)

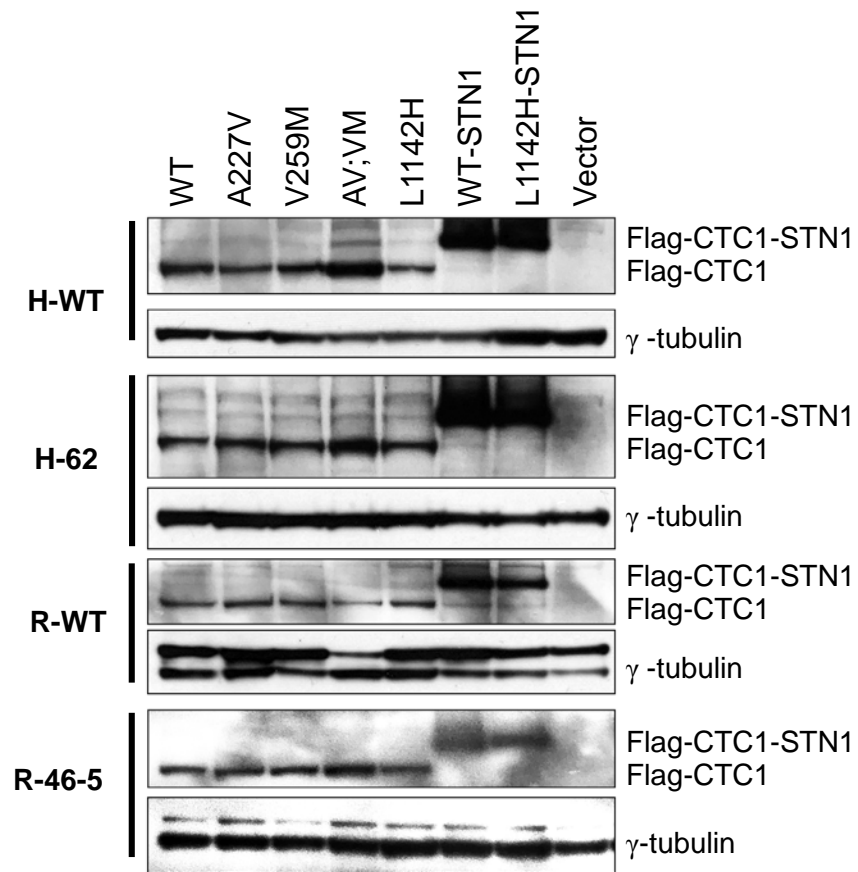

(b)

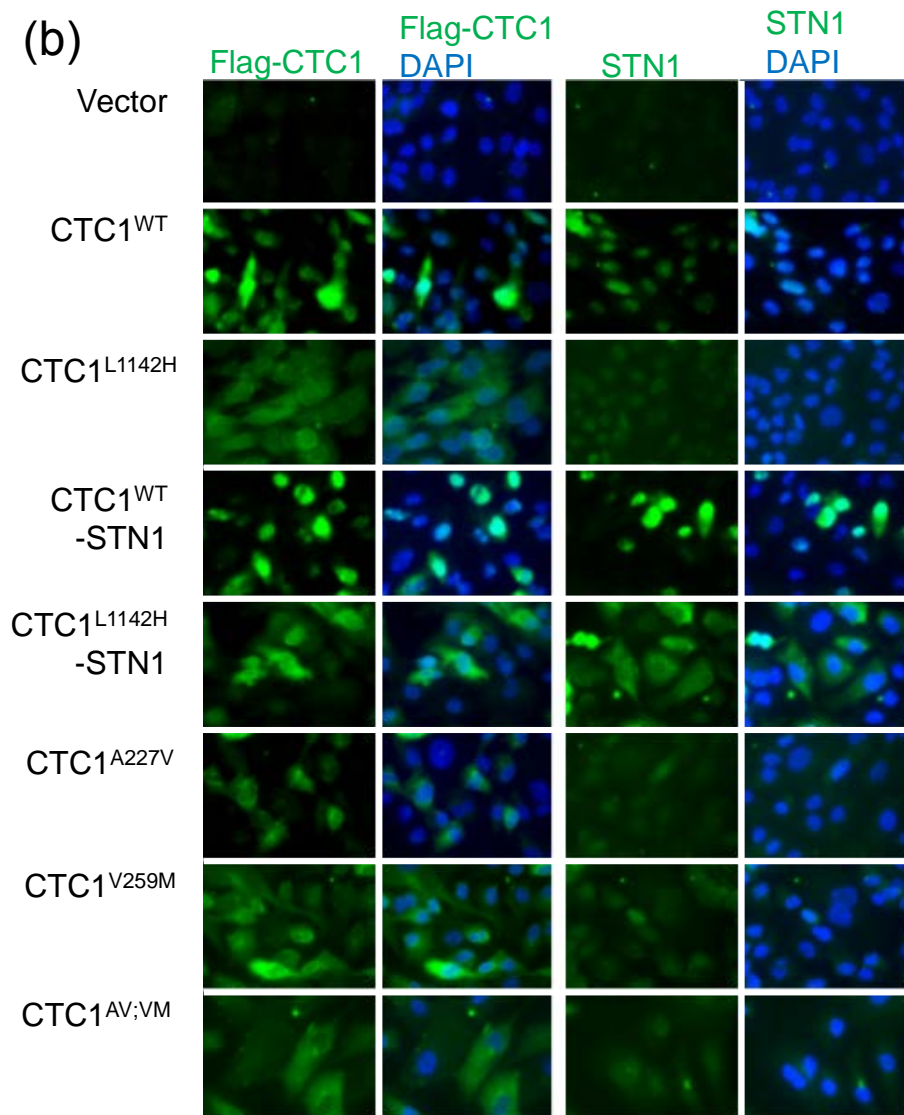

RPE-46-5 (L1142H)

(a)

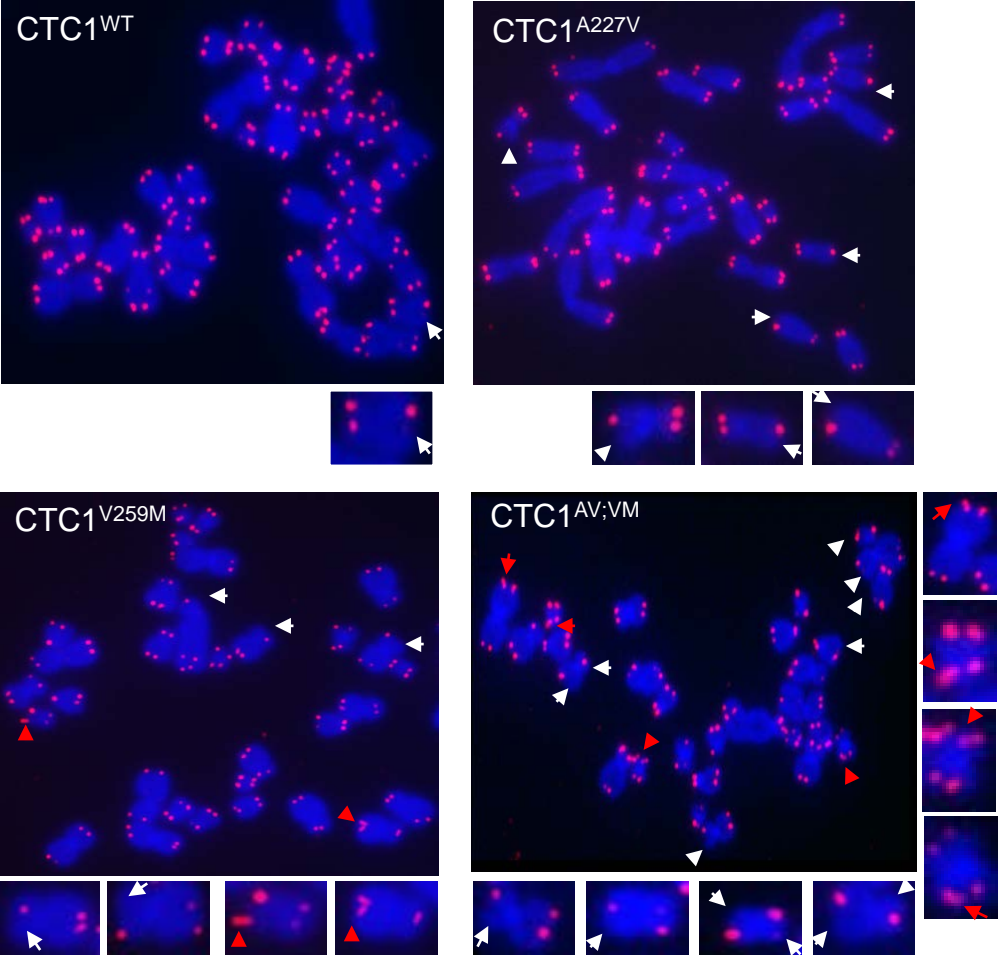

(b)

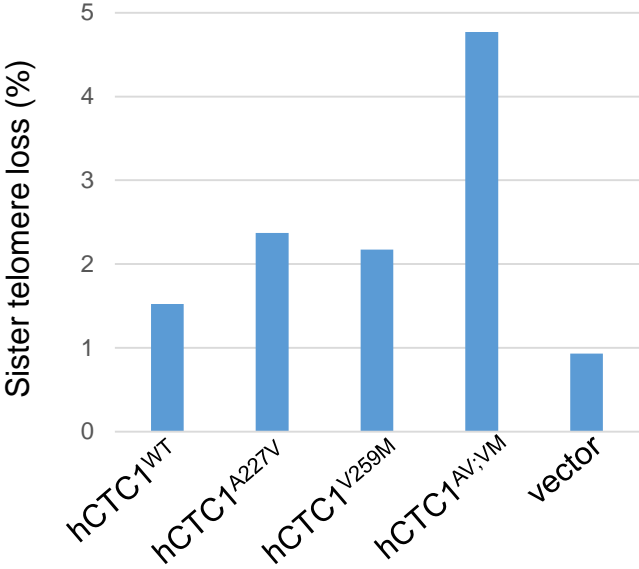

(c)

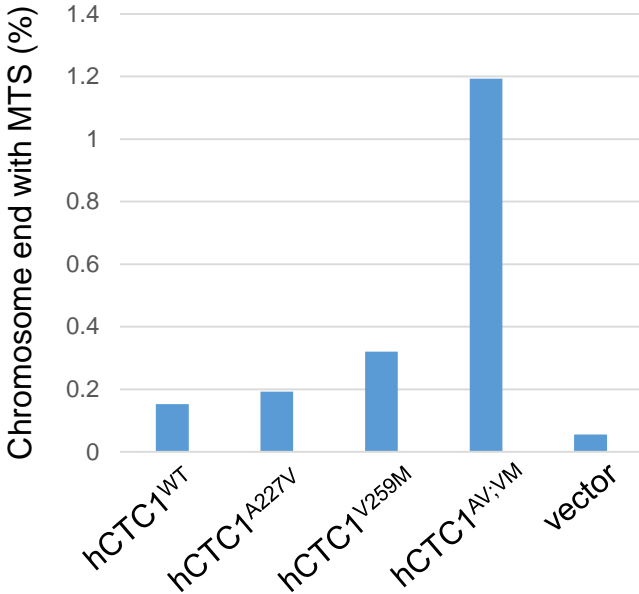

Supplement: Supplementary file 1 [file ACEL-17-na-s001.pdf]
